# The History and Capabilities of Herbal Simples

**Published:** 1893-08-19

**Authors:** W. T. Fernie


### THE HISTORY AND CAPABILITIES OF HERBAL SIMPLES.

By W. T. Fernie, M.D.

(*Continued from page 262.*)

### GARLIC AND THE ONION.

An enduring gastronomic reputation has attached itself to the *chef* who chewed a small clove of garlic, and then breathed lightly over the *plât* he wished to flavour. And the taste of a roast leg of mutton is much improved if a small clove of garlic is first inserted into the knuckle, whilst the joint is served up with white haricot beans. To this powerfully odorous bulb is attributed the singular property that if a morsel thereof is masticated by a man running a race, it will prevent any competitor from getting ahead of him. So the Hungarian jockeys often tie a clove of garlic to their racers' bits, that the horses which run against them may fall back the moment they smell the offensive scent. Nowadays nervous headache will sometimes yield to garlic or to asafœtida, its twin sister. Thus a quaint old charm teaches us :—

> " Give onyons to Saynt Cutlake,
> And garlyke to Saynt Cyryake,
> If ye will shun the headache—
> Ye shall have them at Queenhyth."

Throughout many parts of England, in moist woods, hedges, and meadows, grows the wild garlic, strong smelling, and having one or two broad, large, egg-shaped, bright green leaves. It is often called ramsons (" ramse " being rank of odour), or buck rampe, having flowers of pure white with acute petals. A well-known old saying runs to the effect that to

> " Eat leeks in Lyde (March) and ramsins in May,
> All the year after physicians shall play."

The leaves of ramsons are taken in the low countries, with fish for a sauce.

The Leek is called botanically Allium porrium, a remnant of the Anglo-Saxon " porleac," and it also bears the name of purret. The bulb is stimulating, and expectorant, containing sulphur like its kindred plants. The juice is diuretic, dissolving gravel and earthy phosphates in the urine. A time-honoured distich declares that " lovers live by love, and larks by leeks." For chilblains, sore eyes, and chapped hands, the juice of a leek squeezed out and mixed with cream is a reputed cure. In Wales the leek is dedicated to St. David, and is worn by all persons on his day (March 1st). The Britons in 640, being known to each other by leeks drawn from a garden near the field of action, and worn in their caps, gained a complete victory over the Saxons, who ignorantly turned their arms against their fellow-countrymen, whom they failed to distinguish. Or it may be that the practice of wearing the leek on the first of March took its rise from the custom of Cymortha among Welsh farmers. In some districts of South Wales all the neighbours of a small needy landowner attended on this certain day to help plough his fields, and each one brought a leek with him to make the soup for dinner. An inscription on one of the pyramids shows that leeks were the food of the poor in ancient Egypt, and it has been suggested that the phrase, " to eat the leek," is connected with this fact. Their loss as food was bewailed by the Israelites when journeying through the Desert. In our time they have commended themselves generally as a wholesome spring vegetable. Tusser tells us in his book of " Monthly Husbandry," for March

> " Now leeks are in season, for pottage full good,
> That spareth the milch cow, and purgeth the blood."

In the story of Pyramus and Thisbe reference is made to the beautiful emerald green which the leaves of this esculent exhibit. " His eyes were green as leeks." And the Roman Emperor, Nero, was so fond of leeks that he was called in scorn, " porrophagus."

An amusingly rhymed riddle about the onion runs thus—

> " 'Charge, Stanley, charge ! On, Stanley, on !'
> Were the last words of Marmion.
> If I had been in Stanley's place
> When Marmion urged him to the chase,
> In me you quickly would descry
> What draws a tear from many an eye."

